# Cellulose Triacetate (CTA) Hollow-Fiber (HF) Membranes for Sustainable Seawater Desalination: A Review

**DOI:** 10.3390/membranes11030183

**Published:** 2021-03-08

**Authors:** Takahito Nakao, Yuki Miura, Kenji Furuichi, Masahiro Yasukawa

**Affiliations:** 1 Desalination Membrane Department, Toyobo Co., Ltd., Osaka 530-8230, Japan; 2 Graduate School of Science, Technology and Innovation, Kobe University, 1-1 Rokkodai, Nada, Kobe 657-8501, Japan; 3 Iwakuni Membrane Plant, Toyobo Co., Ltd., 1-1 Nadamachi, Iwakuni, Yamaguchi 740-0033, Japan; 4 Research Center, Toyobo Co., Ltd., 2-1-1 Katata, Ohtsu-City, Shiga 520-0292, Japan; kenji_furuichi@toyobo.jp (K.F.); masahiro_yasukawa@toyobo.jp (M.Y.)

**Keywords:** seawater desalination, cellulose triacetate, hollow fiber membrane, brine concentration

## Abstract

Cellulose triacetate (CTA)-based hollow fiber (HF) membrane is one of the commercially successful semipermeable membranes that has had a long progress since the time the excellent semi-permeable feature of cellulose-based polymers was found in 1957. Because of the reliable and excellent performances, especially for drinking water production from seawater, CTA-HFs have been widely used as reverse osmosis (RO) membranes, especially in arid regions. In this review, recent developments and research trends on CTA-HF membranes for seawater reverse osmosis (SWRO) plants were presented. A flux analytical model, an optimization strategy for chlorine injection without losing salt rejection performance, and a module of current high performance CTA RO membranes along with its plant operation data were updated in this paper. Furthermore, a newly developed CTA-HF membrane for brine concentration (BC) application (called BC membrane) was also addressed. Finally, RO/BC hybrid operation was introduced as an effective SWRO desalination technique that enables minimizing the volume of brine disposal from the RO plant by increasing the recovery ratio and the subsequent amount of produced freshwater, without an additional energy input.

## 1. Introduction

A rapid growth of global population and urbanization especially in developing countries has resulted in a huge demand for fresh water in many regions of the world [1,2,3,4]. Fresh water shortages are becoming a crucial threat for the securing of sustainable water, human health, ecosystems, and economic development [5,6,7]. At present, about 40% of the global population faces severe water scarcity, and it is still expected to increase [7]. Because the total amount of fresh water on the earth remained almost the same despite the increasing demand, seawater desalination for reliable water securing has been sufficiently important as a major water source especially in arid regions. Seawater reverse osmosis (SWRO) has been recognized as a reliable mainstream method in the world because of its lower energy consumption compared with the conventional desalination techniques (such as evaporation) due to the absence of latent heat energy for the phase changing of water [8]. Theoretically, about 1.0 kWh of energy is needed to produce 1 m^3^ of fresh water with the seawater recovery ratio of 50% [9,10,11]. However, about 2.6–8.5 kWh of energy is generally required in the current RO plants [12], and minimizing the energy consumption is still needed. Furthermore, the recent environmental concerns and legal regulations require minimizing the environmental impacts of brine disposal from SWRO plants as well as wastewater treatment plants [13,14,15,16]. For example, the total global installed desalination capacity is 97.2 million m^3^/day while the total global cumulative contracted capacity is 114.9 million m^3^/day as of the middle of Feb 2020 [17], that is, a huge amount of brine (about 140 million m^3^/day) was discharged into the ocean (under an acceptable assumption that its water recovery ratio was 42% [16]). Therefore, brine management has been recognized as an important matter due to the rapid increase in the number of desalination plants. In fact, for example, 22 million m^3^/day of brine (15.5% of the total brine) is produced inland (over 50 km from the nearest coastline) [16], and it creates transportation problems for the brine discharge to say the least. Therefore, the optimization of brine management for the plants via economically viable and environmentally friendly ways will become quite important in the near future [15,18]. For this purpose, both brine volume minimization and the subsequent reuse of the brine as a valuable resource are regarded as promising options because minimizing the brine will reduce the amount of brine as well as increase the water recovery in the RO plant. In addition, the subsequent brine mining can transform waste brine into valuable products [19,20,21]. 

A semipermeable membrane is a key component in the SWRO plants that enables the extraction of fresh water from seawater. Moreover, it also has a huge potential for brine minimizing. Up to now, only cellulose triacetate (CTA)-based and polyamide (PA)-based membranes have been successfully used in the SWRO plants for reliable water securing since the first finding of an outstanding semipermeable membrane of cellulose-based polymers in 1957 by Reid [22]. Cellulose triacetate (CTA)-based hollow fiber (HF) membrane is therefore one of the commercially successful semipermeable membranes that has a long historical development due to its reliable excellent performances especially for drinking water production from seawater. In addition, recently, because of the versatile demands in energy consumption and environmental concerns about brine discharge as mentioned above, CTA-based HFs have caught attention for the usage of different methods, such as forward osmosis (FO) [23,24,25,26], pressure-retarded osmosis (PRO) [11,27,28,29,30,31,32,33] and brine concentration (BC) [34,35] for the emerging technologies. Because their applications require optimum characteristics individually, different strategies for designing the membrane and module are required in spite of using the same CTA material. In this review, the recent development and research trends on the CTA-HF RO membranes are updated. This review covers the developments of CTA-HF membranes, including their historical background, the module configuration, flux theory for the module, and long-time plant operation data. Furthermore, this review provides the development of a CTA-HF membrane specialized for BC applications. These updates are promising, namely in how they will be useful for not only developing the CTA-based membrane and module but also for contributing to solving the brine management problem for sustainable SWRO desalination in the future. 

## 2. Brief Background of the CTA Membrane for Seawater Desalination 

The RO process was firstly proposed by Reid of Florida University in the beginning of 1953 [23,36]. After then, the workers at Florida University announced a cellulose diacetate (CDA) film with outstanding semipermeable performance in which salt rejection was 96% or more, but its water permeability was still very small and not applicable for commercial application [37,38]. However, their pioneering work clearly showed a possibility of conducting a RO process using a semipermeable membrane for seawater desalination. After that, a remarkable progress was achieved by Loeb and Sourirajan of the University of California, Los Angeles (UCLA); they prepared an asymmetric-type CDA membrane that had quite a thinner salt rejection layer and a loose support sublayer using the same material of CDA [39,40]. After this invention, accelerated research on cellulose-acetate (CA)-based materials for practical applications was conducted to improve the membrane performance in the industry [41]. The details in the early history of RO membrane development are available elsewhere [37,41,42,43,44,45]. As CA-based polymers, cellulose triacetate (CTA) [46], cellulose acetate butylate [47], CA blends such as CDA with CTA [45,48], the cross-linked CA [49], and the composite membrane (a thin CA film on a porous support consisting of the other polymer) [50] were demonstrated in addition to the CDA. Towards its commercialization, among them, CTA membranes were well developed by some companies because CTA had a better stability in a wider range of temperatures and pH, higher resistance to chemicals and biological attack, and better membrane performance compared to the initial CDA, even though CTA was more difficult to handle in the membrane preparation than CDA [41]. Then, the preparation conditions such as dope solution composition and post-treatment condition were also further developed and optimized for the applicable industrial production. For example, the dope solution contained CA polymer, acetone, and magnesium perchlorate at −10 °C casted on a glass plate. The casted solution was set for a certain time as an evaporation procedure and then immersed in cold water [39,40]. Since the first method was difficult, an easier preparation procedure with high membrane performance was required in the industry [41]. Although various types of solvent for the dope and coagulation bath were then studied (such as acetone, acetic acid, formamide, chloroform, pyridine, dioxane and its mixtures), these solutions were difficult to handle in the industry. Therefore, polar aprotic solvents such as *N*-methyl pyrrolidone (NMP), dimethylacetamide (DMAc) and dimethylsulfoxide (DMSO) were preferably used as the alternative solvents because of their easy handling in the industrial production.

In addition to the membrane development, subsequent module configurations were also developed, such as tubular [42,51], plate-and-flame [52], spiral-wound [53,54], and HF [55]. Among them, the HF configuration had superior advantages to the spiral-wound in the total membrane area, the packing density, and the module performance if the original membrane performance was the same. The spiral-wound type CA membrane was commercialized by many companies and used as the industry standard through the 1960s to mid-1970s [37,56]. However, CA-based membranes were still not widely used for the large-scale desalination purpose at that time [57]. The CA-based spiral one was mainly used for pure water production for industrial processes and ultrapure water production in the semiconductor industry [57]. After that, in the case of the spiral-wound type, an alternative membrane was found via the pioneering work by Cadotte from 1972 [56,58], which was based on a situ condensation method using monomeric amine and monomeric acid halide. This innovative invention made a rapid shift from the CA-based spiral-wound to the PA-based one because of the preferable features of the PA-based membrane, such as higher flux and higher pH resistance.

With the purpose of achieving seawater desalination in mind, a significant portion of research and development was carried out using a CTA-HF membrane and module by Dow Chemical based on a research contract with the Office of Saline Water in the U.S. Department of the Interior [37]. Their development results of the RO module for brackish water desalination and for seawater desalination were published in 1970 and 1974, respectively [59,60], and the CTA-based HF RO module for low-pressure brackish water desalination was subsequently marketed in 1974 [37]. The research developments for the CA-based HF were also conducted by other companies such as Monsanto, Toyobo, and others, in addition to Dow Chemical. After that, following the early work by Dow Chemical, Toyobo successfully developed a CTA-HF RO module for high-pressure one-pass seawater desalination and subsequently marketed it in 1979 [37,45,61]. The CTA-HF RO module was often used in the fishing industry as a small-scale desalination apparatus on a shipping boat [62]. 

After these efforts, because of the rapid increasing demand for drinking water in the world, RO membranes have been acceptably and widely used for the large-scale seawater desalination purpose since the 1990s; the total global cumulative contracted capacities since 1965 were about 14, 25, and 60 million m^3^/day in 1990, 2000, and 2010, respectively [1]. In the case of the HF type, only CTA is still used for the RO membrane for seawater desalination, unlike the spiral-wound type, because of its competitive total module performance of CTA-based HF membrane module compared with the PA-based spiral-wound one [29].

## 3. Features of CTA-HF RO Module 

### 3.1. CTA-HF Membrane 

#### 3.1.1. Preparation Procedure and Its Characteristics

**Figure 1:**
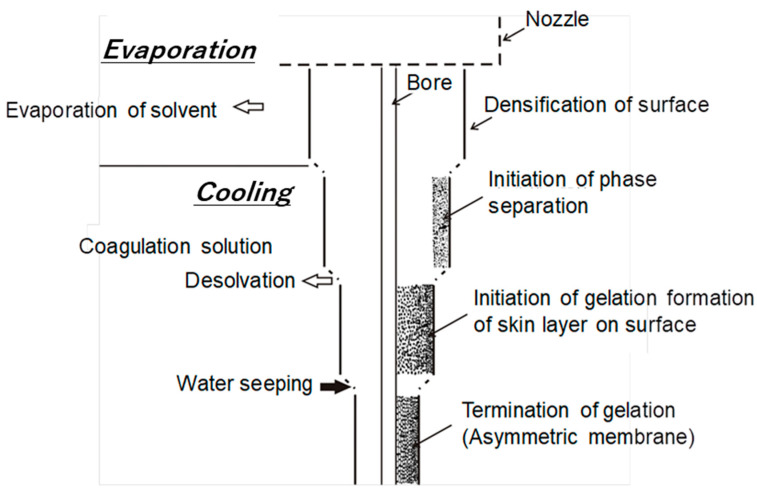
Typical diagram for preparation of an asymmetric membrane.

**Figure 2:**
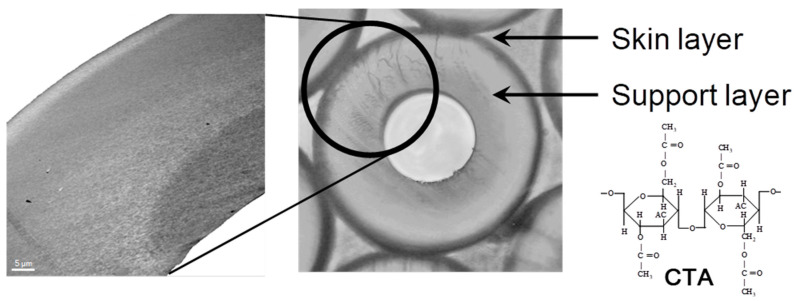
Image of hollow fiber (HF) membrane in cross-sectional structure and TEM image.

CTA-HF RO membranes are generally prepared by spinning a solution of a CTA polymer, followed by soaking and annealing. The optimization of the preparation condition, such as using spinning technology of high concentration polymers, micropore controlling technology in manufacturing, and post-treatment by high temperature annealing, made it possible to increase the permeate water flux of the CTA-HF membrane without additional steps [37,38]. A typical illustration for HF preparation procedure for an asymmetric membrane is shown in Figure 1. Highly-controlled spinning technology provided a well-designed morphology of the HF with an outer dense layer for selective water permeation and a relatively gradient porous structure of the HF along the thickness direction from the outer to inner diameters. Figure 2 shows the optical and transmission electron microscopic images (OM and TEM) of the CTA-HF RO membranes. In the TEM image, the CTA-HF RO membrane has roughly three layers (the outermost layer in white is the dense separation layer) with gradient morphology. Water- and salt-selective permeations strongly depend on the outer dense layer, and the porous support layer provides the pressure resistance of the HF membranes. Therefore, this gradient porous morphology along the outer dense layer and the porous inner support layer provided the CTA-HF RO membranes with outstanding pressure resistance retention without additional supports. Moreover, the gradient morphology will be formed even within a dense thin layer because the dense layer in the TEM image is a lot thicker than expected from the theoretical permeability of the HF membrane estimated from its thickness in the TEM image. 

The HF dimensions with a fine HF structure are also important characteristics to give not only a pressure resistance of HF but also water permeation performance. The outer and inner diameters of CTA-HF RO membranes are designed to be about 140 μm and 55 μm, respectively. The suitable dimensions of the HF are controlled by the preparation condition and the nozzle dimensions in the spinning process.

#### 3.1.2. Chlorine Resistance

A membrane clogging (fouling) caused by fouling substances (foulants) is a major problem in the daily operations in the seawater RO desalination plants because it significantly deteriorates the performance of membrane permeability and plant productivity [63]. Because the scaling is generally controlled by adjusting the pH of seawater and its recovery ratio, the most serious fouling problem in the RO plant is microorganism-based biological fouling [64]. Microorganisms adhere to the RO membrane that utilizes the foulants (such as organic substances attached onto the membrane surface), and the various microorganisms then proliferate, forming a biofilm [65]. Once a serious biofilm is formed on the RO membranes, the performance recovery would become too severe; therefore, precautions are generally taken using chemical treatments such as chlorine injection for sterilizing and suppressing the proliferation of microorganisms. For this purpose, chlorine tolerance of the membrane is an important characteristic for a stable and reliable RO plant operation without losing the salt rejection performance of the membrane. Because of CTA having less chlorine reactivity than PA [66], the CTA RO membranes have a higher chlorine resistance than that of polyamide-based (PA) RO membranes, and this enables a reliable and stable RO operation without any biological fouling problems for a long period by implementing a simply controlled intermittent chlorine injection (ICI).

#### 3.1.3. CTA Membrane Characteristics Compared to PA-Based Membrane

In addition to the development of a CTA-based RO membrane, PA-based RO membranes also had a period of rapid development, starting from the time of Cadotte’s pioneering work in 1972 [56,58], and they were successfully used for seawater desalination purposes. Compared to the CTA-based membrane, PA-based membrane has superior performance characteristics, such as higher flux per membrane area as well as higher chemical and pH resistances. In particular, the unique preparation procedure of PA-based membranes via interfacial polymerization on the porous support layer allows for a very thin selective layer formation, which results in much higher water permeability than the CTA-based membrane. On the other hand, at the same time, the two-step membrane preparation procedure of a PA-based membrane (porous support layer preparation and subsequent interfacial polymerization) might impede the mass production of the PA-based membrane applicable for the HF configuration, especially the outer-selective layer type of the PA-based membrane [67]. Although the CTA-based membrane has lower water permeability than the PA-based membrane, the simple preparation procedure of the CTA-based membrane allows for a HF configuration and a more highly effective membrane area compared to the PA-based membrane. Consequently, the resulting module performances of CTA and PA-based membranes have been in competition with each other in the viewpoint of module performance.

The other difference between CTA and PA-based membranes is the stability of the membrane, such as chlorine resistance and membrane compaction resistance. As for the chlorine resistance, in the case of CTA-based membranes, because the CTA is an environmentally friendly polymer (biomass-based plastic and biodegradable), moderate chlorine disinfection is generally recommended to prevent the excessive degradation of the membrane during the operation, whereas chlorine disinfection is generally not recommended in the case of PA-based membranes because chlorine exposure causes performance deterioration of the PA-based membrane [68]. As for the compaction resistance, the compaction tolerance of the CTA-based membrane is less than that of PA-based membrane, which causes a flux performance reduction of the CTA-based membrane during the initial operation stage [69]. On the other hand, the PA-based membrane has higher compaction resistance than the CTA. To overcome these problems in both PA and CTA-based membranes, the recent studies have demonstrated an additional blending strategy using siloxanes [70], carbon nanotubes [71], and nanoparticles [72] for PA-based membrane with chlorine tolerance, as well as a reinforcing strategy using nonwoven fabric [73] for CTA-based membrane with compaction tolerance.

### 3.2. Module for CTA-HF Membranes

#### 3.2.1. Module Design

**Figure 3:**
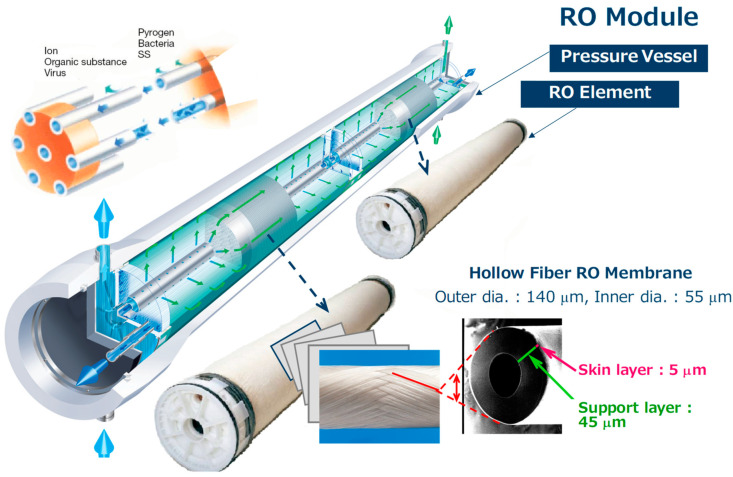
Structure of cellulose triacetate (CTA) reverse osmosis (RO) membrane module.

CTA-based membranes are easily applicable for a HF configuration because of its simple preparation procedure as mentioned above. A HF-type RO membrane module comprises a HF-type RO membrane element and a pressure vessel as shown in Figure 3. This HF membrane element has a structure in which a large number of HFs are arranged around a central pipe with holes; both ends are bonded with epoxy resin, and either one or both ends are cut to create open surfaces. The feed water is supplied from the central pipe through the holes (green arrows in Figure 3). Only pure water permeates through the HF membranes entering inside the HF, and then the permeate water is collected from an outlet at one or both ends (blue arrows in Figure 3). The feed water that did not permeate through the HF membranes (concentrated water) is collected from another outlet at the pressure vessel.

#### 3.2.2. Cross Arrangement of HFs 

In seawater desalination, when the seawater concentration becomes higher, the osmotic pressure becomes higher also, making the operating pressure required for the water production higher. Generally, the recovery ratio (ratio of pure water extracted from seawater) by the reverse osmosis membrane method is 40–60%. In the reverse osmosis method, a concentration polarization layer with a high salt concentration forms on the membrane surface. Therefore, it is important to keep the concentrated seawater flowing uniformly on the membrane surface to prevent an increase in seawater concentration near the membrane surface, which will result in a decrease in water permeability. For this reason, the arrangement of HF membranes around the feed pipe has been optimized to realize a uniform seawater flow. Specifically, the CTA-RO membrane, which is a very fine fiber with an outside diameter of about 140 μm, is crossly wound around the central pipe to create some space between the fibers so that the seawater can flow evenly [38]. This cross-wound configuration can lower the seawater concentration near the membrane surface and enable the entire membrane surface to be effectively used.

#### 3.2.3. Optimum Permeability (Lower Distributed Flux)

**Figure 4:**
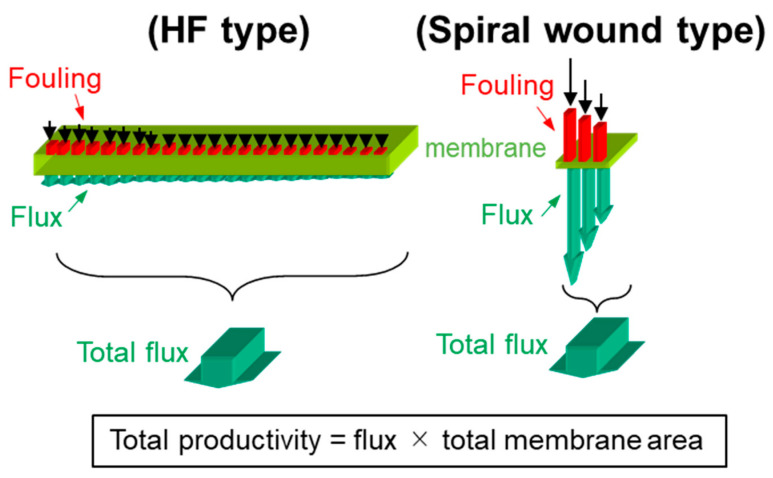
Comparison of fouling risk in membrane type [28].

In a CTA-HF RO membrane element, millions of HFs are wounded into the element by a cross-wound technology. Compared to the spiral-wound RO membrane element, CTA-HF membranes has lower water permeability than the spiral-wound (polyamide-based) membrane. However, the CTA-HF membrane element has about 7–10 times larger surface area than that of spiral-wound element, and so the total module performance can be competitive [28,29]. In this case, a lower membrane flux of the CTA membrane has beneficial advantages in terms of a lower membrane fouling risk and less concentration polarization than those in the spiral-wound cases as shown in Figure 4 [28]. Consequently, the membrane cost per its effective membrane area (cost/m^2^) of the CTA-HF RO membrane is about 7–10 times cheaper than that of the spiral-wound RO membrane, resulting in a highly efficient usage under a lower operational stress.

### 3.3. Flux Theory for CTA-HF RO Module 

#### 3.3.1. FCP Model

To predict the CTA-HF RO module performance, a friction concentration polarization (FCP) model [74,75] is often used as an analytical model. The FCP model is an initial analytical model of a HF RO membrane module in which a pressure loss inside the HF is considered according to the Kimura-Sourirajan membrane transport equation [76]. In a FCP model, the HF’s water flux across a semipermeable membrane is described as follows:

(1) $$J_{w}=A\left(P-\Delta \pi -P_{bp}\right)$$

where *J*_w_ is the water flux; *A* is the pure-water permeability; *P* is the average of applied pressures at the shell (seawater) side; Δ*π* is the average osmotic pressure difference; and *P*_bp_ is the average of pressures at the bore (permeate) side. The water flow through the membrane is generally not constant along the HF length because the *P*_bp_ changes along the fiber length as follows [74,77]:

*P*_bp_ = *Le OD Q*_w_ (*Lts* + *Le*/3)/*ID*^4^ (2)

where *Le* is the effective HF length; *Lts* is the tube sheet length; *Q*_w_ is the water flow; *OD* is the outer diameter; and *ID* is the inner diameter. Thus, to increase the water flux of the HF membranes, having a lower *P*_bp_ is important in order to increase the *J*_w_ by fixing the *OD* and *Q*_w_. The *P*_bp_ can be decreased by the following ways:

1. Reducing the effective fiber length, *Le*;
2. Increasing the fiber diameter, *ID*;
3. Reducing the tube sheet length *Lts*.

**Figure 5:**
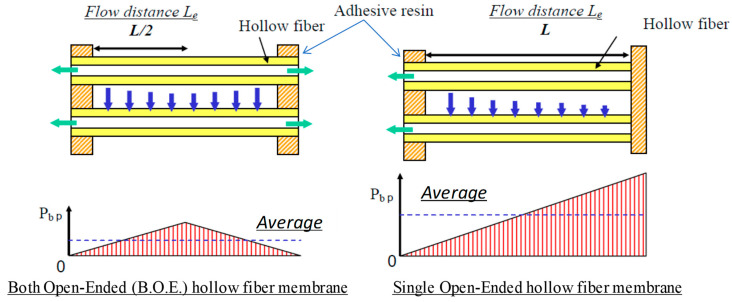
Comparison of fouling tendency in membrane type [38].

However, the above approaches also decrease the total effective membrane area of the module and would decrease the total module performance. Therefore, an optimization of the module design is necessary in order not to lose the total performance of the module. One of the effective approaches is the both open-ended configuration (BOE) as shown in Figure 5. Compared with the single open-ended configuration (SOE), the average *P*_bp_ becomes lower, which results in higher water permeate flux. In the case of the BOE type, the location of the maximum *P*_bp_ is about the half of the HF length, and its value becomes lower than that of the SOE type as shown in Figure 5 [38].

The permeate water quality (i.e., salt rejection) and the concentration of the permeate water flow, *C*_p_, can be described as follows:

(3) $$J_{s}=B\Delta C$$

(4) $$C_{p}=\frac{J_{s}}{J_{w}}$$

where *J*_s_ is the solute flux through the membrane; *B* is the membrane permeability coefficient for salt; and Δ*C* is the solute concentration difference between the feed and the permeate sides. Because the *J_w_* of the BOE type module increases, the *C*_p_ is reduced if the salt passage is the same. Therefore, the lower applied pressure, *P*, can be used, compared with the SOE module case [78].

#### 3.3.2. CFD/FCP Model

Although the FCP model for HF module has been validated by a wide range of actual data, it still has some drawbacks (assumptions for the calculation) [79], namely that (1) the shell side seawater stream with axial direction is neglected; and (2) the feed side solution from the distribution pipe of the HF module is uniform along the module axis.

Therefore, in order to further consider the fluid dynamics in the HF module, a new integrated model based on computation fluid dynamics (CFD) with FCP (CFD/FCP model) was recently proposed [79]. The CFD/FCP model can calculate the flow and mass transfer throughout the entire module with appropriate boundary conditions. For example, the feed flow rate at the inlet of the distribution pipe and the applied pressure can be set as boundary conditions, respectively. The equations for the FCP model are implemented using user-defined function (UDF) in the commercial CFD software ANSYS Fluent R14.0 (Fluent) to account for the mass balance through the membrane and flow pressure loss in the HFs. The simulated domain of the CFD/FCP model includes the distribution pipe, the HF bundle, and the clearance between the HF bundle and the pressure vessel. The zone of HF bundle is treated as a porous zone with a laminar flow due to the very slow fluid velocity. In the other zones, turbulence equations are solved in addition to the Navier–Stokes equation.

The calculation procedure of CFD/FCP model is as follows:

1. Set the module operating condition as boundary conditions.
2. Set appropriate initial conditions for the inside and outside of HFs.
3. Calculate the pressure, velocity, and concentration distribution outside the HFs using Fluent.
4. Consider the shell side pressure gradient in the HF bundle ∇*P*_B_ using the Ergun equation as follows:

(5) $${\left(\nabla P_{B}\right)}_{j}=\frac{150{\left(1-\varepsilon \right)}^{2}}{\varepsilon^{3}}\frac{\mu_{B}}{d_{eff}^{2}}V_{j}+\frac{1.75\left(1-\varepsilon \right)}{\varepsilon^{3}}\frac{\rho_{B}}{d_{eff}}V_{j}^{2}\;\;\;\;\;\;\;\;\left(j=r,z\right)$$

where *ε* is the porosity of the HF bundle; *ρ*_B_ is the density of bulk solution; *d*_eff_ is the effective surface diameter, i.e., *d*_eff_ = 1.5 *d*_0_ (*d*_0_: outer diameter of HFs); *μ*_B_ is the viscosity of the bulk solution; *V*_j_ is the velocity component of *j*-direction; and *r* and *z* represent the radial and axial coordinates, respectively. Subscript *B* means the shell side.

5. Calculate the volumetric permeate flux (*J*_V_) using Equations (1), (3), (6)–(8) implemented in the UDF and pass it to Fluent as a source term of the mass balance equation in the HF bundle. The concentration polarization coefficient at membrane surface Φ is described by Equations (6)–(8):

(6) $$\Phi =\frac{C_{m}-C_{p}}{C_{b}-C_{p}}=\exp\left(\frac{J_{V}}{k}\right)$$

(7) $$J_{V}=\frac{J_{w}-J_{s}}{\rho_{p}}$$

(8) $$C_{p}=\frac{J_{s}}{J_{V}}$$

where *C*_m_ and *C*_b_ are the concentration of membrane surface and bulk, respectively; *k* is the mass transfer coefficient; and subscript *p* means the permeate side.

6. Calculate the bore side pressure gradient, *dP_p_/dz*, caused by the membrane-permeate water inside the HFs using the following Hagen–Poiseuille equation, and then update the pressure distribution in the HFs.

(9) $$\frac{dP_{p}}{dz}=\frac{128\mu_{p}Q_{p}}{\pi d_{I}^{4}}$$

where *Q*_p_ and *d*_I_ are the flow rate of the permeate side and the inner diameter of the hollow fiber, respectively.

7. Repeat the steps 3 to 6 until the pressure, velocity, and concentration distribution in Fluent are converged to the steady state.

**Figure 6:**
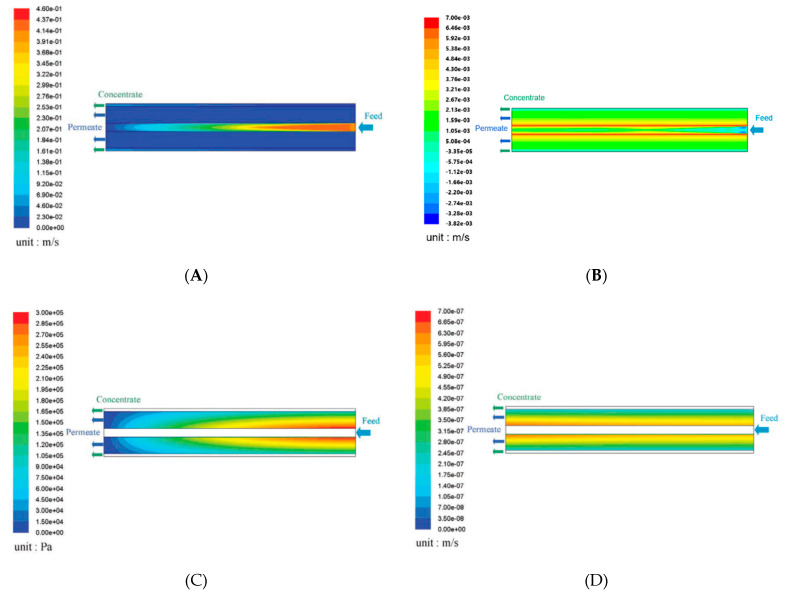
Simulation results by the computation fluid dynamics (CFD) using friction concentration polarization (FCP) model (i.e., CFD/FCP) for the single open-ended configuration (SOE) module. (**A**) Magnitude of shell side velocity in m/s. (**B**) Radial component of shell side velocity in m/s. (**C**) Bore side pressure in Pa. (**D**) Volumetric permeate flux in m/s. In the simulation, the inlet flow rate was 0.475 kg/s; salinity was 35 kg/m^3^; the pressure at the outlet of the permeate side was fixed to be ambient pressure; the pressure at the outlet of the concentrated side was set to 5.5 MPa with a recovery ratio of 30%. Previously published as part of The International Desalination Association (IDA) World Congress Proceedings, San Diego 2015 [79].

Figure 6 shows the simulation results by the CFD/FCP model for the SOE module configuration in terms of the magnitude of the shell side velocity, the radial component of shell side velocity, the bore side pressure, and the volumetric permeate flux. In Figure 6, the HFs at the right side of the module are not opened and sealed by the resin, and the concentrated brine goes out of the module from the clearance between the HF bundle and the pressure vessels as shown in Figure 3. In the CFD/FCP model, the flow rate of the feed liquid at the inlet of the feed pipe was set as a boundary condition, and then the average Reynolds number at the inlet of the feed pipe was estimated at about 8,000 (turbulent flow). The flow velocity showed a maximum value at the feed side inlet. The feed side flow velocity along the radial direction of the module was almost uniform along with the axial direction of the module, that is, it was confirmed in the previous assumption in the FCP model. The maximum bore side pressure was 0.3 MPa, which occurred near the inlet of the feed side. The distribution of the volumetric permeate flux *J*_v_ was almost uniform along the axial direction of the HF module, but it slightly decreased because the pressure inside the HF decreased toward the downstream; therefore, the permeate flux, *J*_v_, slightly increased near the single open-ended side.

**Figure 7:**
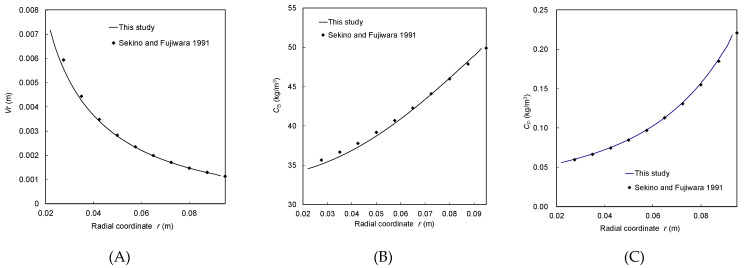
Comparison of the simulation results of radial profile between the conventional FCP model (dots) and CFD/FCP model (lines) for the SOE module configuration at the center of HF bundle. (**A**) The radial flow velocity; (**B**) the salt concentration outside the HF; (**C**) the salt concentration inside the HF for the SOE module configuration.

Figure 7 shows a comparison between the simulated results by the conventional FCP model (dots) and the FCP/CFD model (lines) in terms of the radial flow velocity, the salt concentration outside the HF, and the salt concentration inside the HF for the SOE module configuration. The results clearly indicate that the FCP simulation results (7 parts in the module) well agree with the FCP/CFD simulation results in the whole module, that is, the conventional FCP model was sufficient to predict the module performance in spite of some assumptions mentioned above.

**Figure 8:**
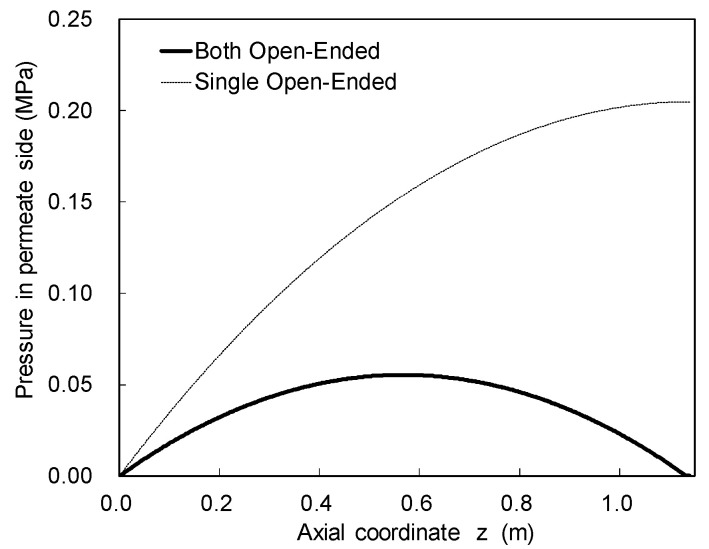
Bore side pressure distribution along the axial direction when using HF with an outer diameter (OD) of 160 μm; operating pressure was 5.5 MPa; 3.5% NaCl was used as feed.

The FCP/CFD model was also adopted to predict the module performance with the BOE configuration. In this case, although the bore side pressure distribution can be obtained by the Poiseuille equation, the boundary conditions are different between SOE and BOE In the case of SOE, its relation between the upstream (close-ended) and the downstream (single-open-ended) is clear. Therefore, if the pressure at the downstream is given as a boundary condition, the bore side pressure distribution can be easily calculated by integrating the Poiseuille equation from downstream to upstream. On the other hand, in the case of BOE, the pressures at the both-open-ends are given as a boundary condition in accordance with the same manner. However, a position of the maximum bore side pressure, which becomes a watershed of the permeate water flow, is unknown. Since this position depends on certain properties, such as the flux behavior, HF length, and so on, a convergence calculation should be adopted to search for the watershed position in the BOE case. Figure 8 shows a comparison of the simulated results between the SOE and BOE configurations. The maximum bore side pressure of BOE (0.05 MPa) was about four times lower than that of SOE (0.2 MPa). Therefore, this difference can work as a driving force for water permeation.

**Figure 9:**
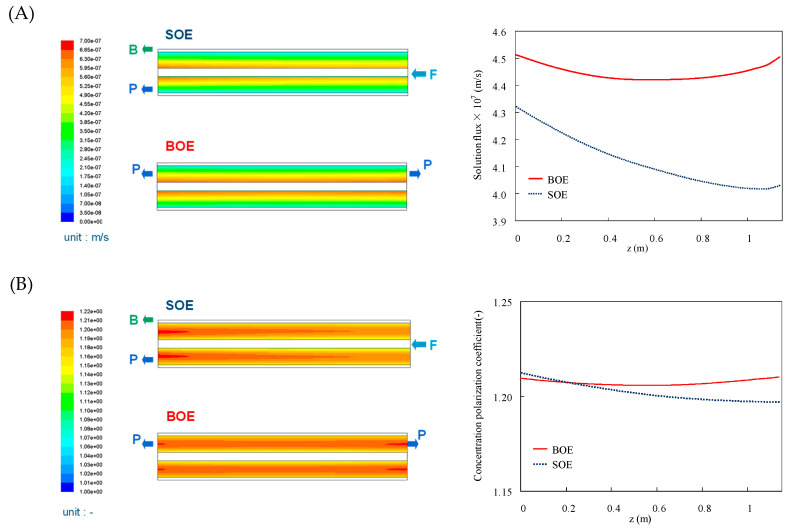

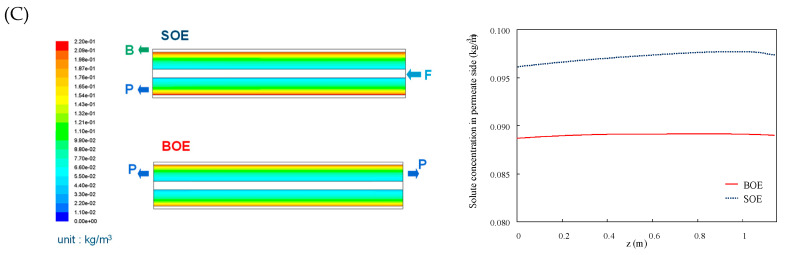
Simulated results by CFD/FCP model. (**A**) Volumetric permeate flux; (**B**) concentration polarization coefficient; (**C**) solute concentration inside the HF and those along the axial (*z*) direction.

Figure 9 shows the simulated results for the BOE and SOE configurations in terms of volumetric permeate flux *J*_v_, the concentration polarization coefficient, and solute concentration of the permeate. In the case of BOE, the feed and brine flows are same with the SOE case, but the permeate water can go out at the both HFs’ ends. In both cases, the highest *J*_v_ appeared at the open-ended parts where the pressure difference between the inside and outside of the HF becomes the largest. In the case of BOE, because the effective pressure difference was larger than that of the SOE case at the whole membrane, all *J*_v_ values became larger than those of SOE cases. On the other hand, the larger *J*_v_ in the BOE also subsequently induces the increase of the concentration polarization coefficient, *Φ*, compared with the SOE cases. However, despite the increase of *J*_v_, *J*_v_/*k* is moderate because the mass transfer coefficient, *k*, also increases when increasing the feed flow rate in the module at the same recovery ratio. Therefore, the maximum value of the *Φ* at the open-end is still larger in SOE than in BOE. The salt concentration (*C*_p_) inside the HF is also estimated from *J*_v_ and the salt flux (*J*_s_). As expected, the *C*_p_ decreases and the water quality of the permeate water can be improved when compared with SOE cases.

**Figure 10:**
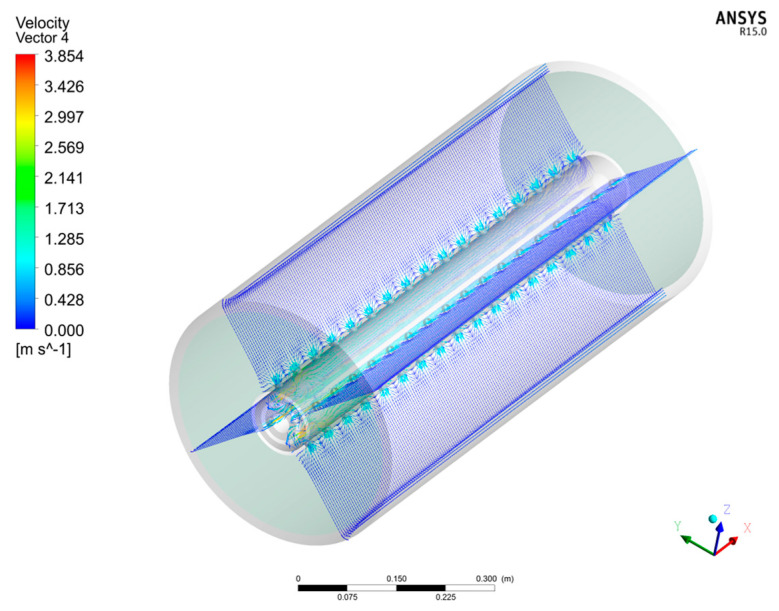
3D vector field of shell side velocity in the HF bundle considering the detailed structure of module.

As introduced above, in addition to the conventional FCP model, the CFD/FCP model is also a useful analytical model to estimate the HF module performance. Moreover, in addition to the above 2D simulation, the FCP/CFD model can also be applied to 3D simulation as shown in Figure 10, which enables an estimation of detailed flow behavior inside the 3D HF module for more precise designing of the module.

## 4. Seawater RO Plant Operation Using CTA-HF RO Module 

### 4.1. Clorine Injection for Successful Long-Term Operation

#### 4.1.1. Optimization of ICI Condition

Chlorine is also used as an important disinfection agent to safely remove the infection risk from pathogenic microorganisms such as cholera, dysentery, and typhoid fever. On the other hand, the usage of chlorine also has adverse features, such as toxicity, carcinogenic of the resultant disinfection by-products (DBP) such as trihalomethane, and subsequent potential environmental risks. Therefore, although some alternative agents such as chlorine dioxide [80,81,82,83,84], chloramine [81,82,85], and ozone [81,82,85] have been studied to reduce those adverse impacts, in any case, a minimization of the usage of disinfection agents under a precise monitoring system without losing reliable water in order to secure safe water quality is quite important. 

In SWRO desalination plants, stable and reliable operations are quite important to ensure the membrane’s lifetime without undesirable additional high pressure caused by membrane fouling. A common method of chlorine injection in the SWRO plants is to inject chlorine into seawater as pretreatment and then to add sodium metabisulfite (SBS) to reduce the chlorine before feeding the seawater into the RO module. This is because the continuous excessive chlorine injection will accelerate the deterioration of membrane performance due to the oxidation of the membrane, even in the case of chlorine-tolerant CTA RO membrane. Therefore, when feeding the chlorine to the CTA RO membranes, the SBS addition is intermittently stopped. The performance deterioration rate of the membrane mainly depends on the chlorine concentration, pH, and temperature [86,87]. In addition, previous literature also indicate that a catalytic action of heavy metals such as iron, cobalt and copper also accelerates this oxidation rate [86]. Therefore, in order to maintain a stable membrane performance for a long time, the ICI system [86] is adopted as a basic method in all the SWRO plants using CTA-HF RO membranes.

**Figure 11:**
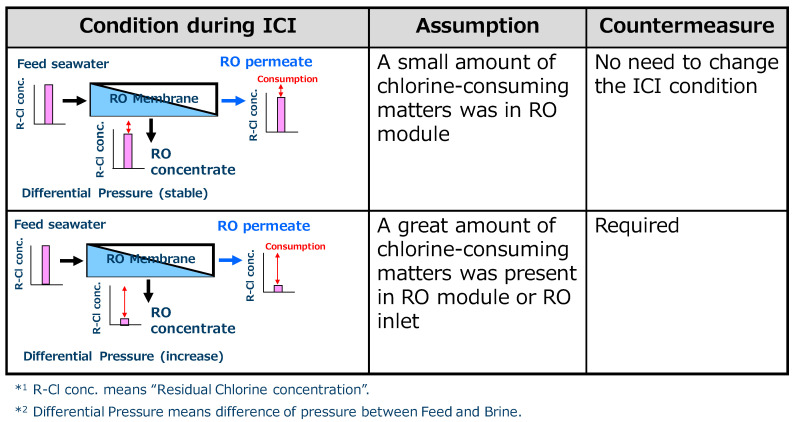
Relationship between intermittent chlorine injection (ICI) condition and chlorine consumption inside the RO module [89].

To minimize the chlorine injection, the chlorine concentration and its injection frequency are important factors for designing an ICI system in a desalination plant. For this objective, the growth rate of microorganisms in the seawater and the bactericidal effect of chlorine were analyzed [88]. In the study, to set the optimum chlorine concentration, the chlorine residue at the outlet of the RO module was focused on. If there are substances such as organic substances and microorganisms as the chlorine-reducing agents that consume chlorine in the feed seawater and RO module, the chlorine consumption during ICI becomes high. The chlorine consumption can be easily calculated from the difference of the chlorine concentration between the inlet and outlet as shown in Figure 11 [89]. If the chlorine consumption is low, the ICI condition can be set as milder. On the other hand, if the chlorine consumption is high, the differential pressure would rise faster than predicted due to biofouling and organic fouling, and more severe ICI conditions must be set.

**Figure 12:**
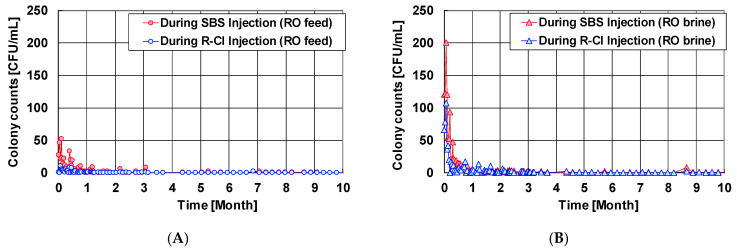
Colony counts results of (**A**) feed seawater and (**B**) RO brine during ICI. The target residual chlorine concentration was 0.1 to 0.2 mg/L with the frequency of chlorine injection at 3 times per 24 h where each time was 1 h. Data from [89,90].

Figure 12 shows the verification results of the bactericidal effect by the ICI method. The target residual chlorine concentration in the RO membrane module during ICI is 0.1 to 0.2 mg/L, and the frequency of chlorine injection was 3 times per 24 h where each time was 1 h. Figure 12 shows that almost no bacteria were detected in the feed seawater and the concentrated brine during the operation period by the colony count measurement. Therefore, the growth of microorganisms in the RO membrane module was effectively suppressed by the ICI method. Furthermore, the differential pressure did not increase abnormally during the stable pure water production with sufficient water permeability and quality. Based on these results, a stable operation can be realized by optimizing the ICI conditions in accordance with the feed water conditions, such as biological activity and concentrations of various organic substances. 

**Figure 13:**
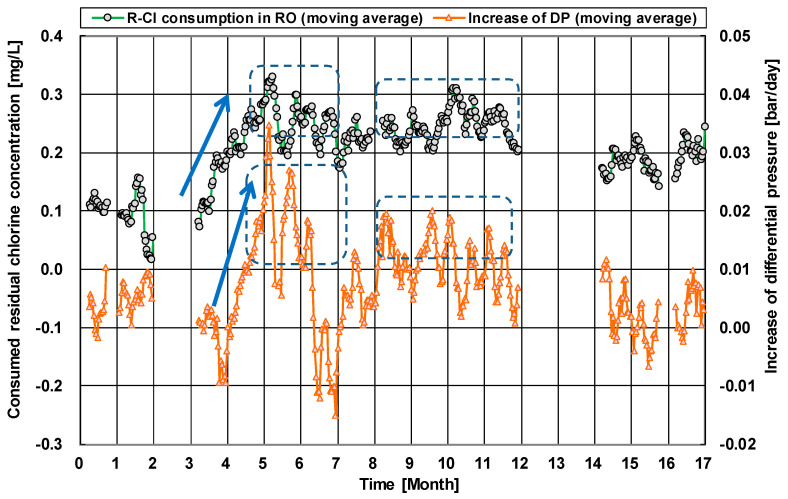
The differential pressure (DP) of the RO module used in a seawater reverse osmosis (SWRO) plant at the Arabian Gulf, and the tendency of chlorine consumption in the RO module during ICI. The target residual chlorine concentration was set to 0.5 mg/L, and its injection time and its frequency were set to once every 24 h where each time was 1 h. Data from [90].

Figure 13 shows the increasing rate of the differential pressure (DP) of the RO module used in a SWRO plant at the Arabian Gulf, and the tendency of chlorine consumption in the RO module during ICI. Here, the chlorine consumption (y-axis in Figure 13) was calculated from the difference in the chlorine concentrations between the inlet (set as 0.5 mg/L) and outlet. The chlorine injection time and its frequency were set to once every 24 h where each time was 1 h. Interestingly, when the chlorine consumption in the RO module increased, the increasing rate of the DP became relatively fast (see the period of the 3rd to the 12th month). On the other hand, when the chlorine consumption during ICI decreased, the increasing rate of the DP was slow (see the 2nd month and the period of the 14th to the 17th month). During the operation, it was already confirmed that almost no bacteria were detected in the feed seawater by measuring the bacteria counts, which means the continuous chlorine injection in the pretreatment system in the RO system (coagulation-sedimentation and double-layer sand filtration) has been an effective bactericide, and the chlorine consumption was due to chlorine-reducing agents only in the RO module. Although some literature suggested that chlorine disinfection was not sufficient to prevent biofouling [91], in these cases, the ICI condition might not have been appropriate. For example, if the chlorine is not detected at the outlet during disinfection, microorganisms may survive and subsequently grow on the surface of the RO membrane. In Figure 13, a sufficient residual chlorine concentration (0.15 to 0.4 mg/L) was continuously detected at the outlet of the RO brine during the 17-month operation with ICI treatment because the chlorine consumption was about 0.1–0.35 mg/L in Figure 13 and 0.5 mg/L at the inlet. However, the DP sometimes increased due to the chlorine consumption in the RO module. Therefore, this increase in the DP was not caused by the formation of biofilm but by the accumulation of organic/inorganic substances in the RO module. Therefore, the ICI conditions should be further optimized based on the quality of the feed water to control the contamination by organic matters and biofouling growth. There are some additional approaches to controlling membrane fouling, such as a further optimization of ICI conditions and the implementation of continuous chlorine injection (CCI) for a short period [92]. However, it should be considered that excessive chlorine injection may shorten the membrane life, even in the case of CTA membranes [86].

#### 4.1.2. Effect of Chlorine Exposure on the Permeate Water Quality

**Figure 14:**
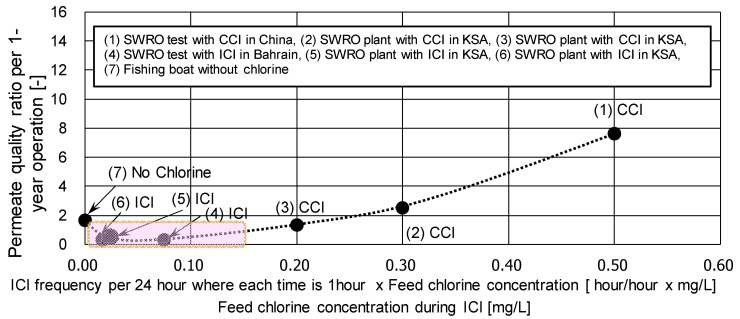
Permeate water quality per 1 year operation in SWRO desalination plants. Data from [90].

Figure 14 shows the effect of chlorine exposure strength (injection frequency times chlorine concentration) on the quality of the permeate water in the various RO desalination plants. This graph clearly indicates that when the chlorine exposure strength is high, the increasing rate of the permeated salt concentration during operation tends to increase (see (1), (2), and (3) in Figure 14.) A possible reason for this deterioration of the permeate water quality is the deterioration of the CTA polymer due to the chemical damage caused by the interaction of chlorine and heavy metals [86]. In addition, even in the case of no chlorine injection condition (see (7) in Figure 14), a slight increase of the permeated salt concentration was also observed, which would be due to the biological damage of the CTA polymer. However, if the conditions of chlorine injection are appropriate (where chlorine exposure strength is from about 0.02 to 0.15), the CTA RO module will not be damaged both chemically or biologically, and there would be stable permeate water quality as shown in Figure 14 (4), (5), and (6). Therefore, it is important to select the appropriate ICI conditions depending on the feed water quality.

### 4.2. Seawater Desalination Plant Data Using CTA RO Module

#### 4.2.1. Jeddah 1 Phase II Plant

**Figure 15:**
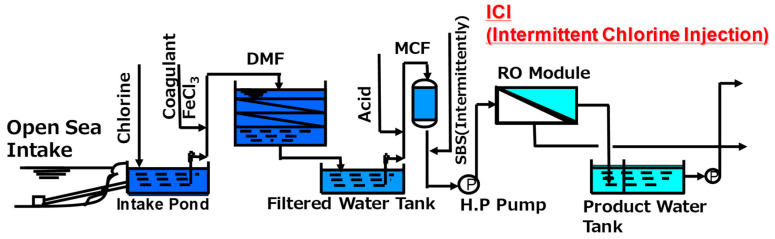
Seawater RO desalination system in Jeddah 1 phase II plant.

In Jeddah 1 phase II plant, a combination of HF RO membranes and the ICI method is adopted and has operated from March 1994 [93]. The schematic illustration of the RO plant system is shown in Figure 15.

**Figure 16:**
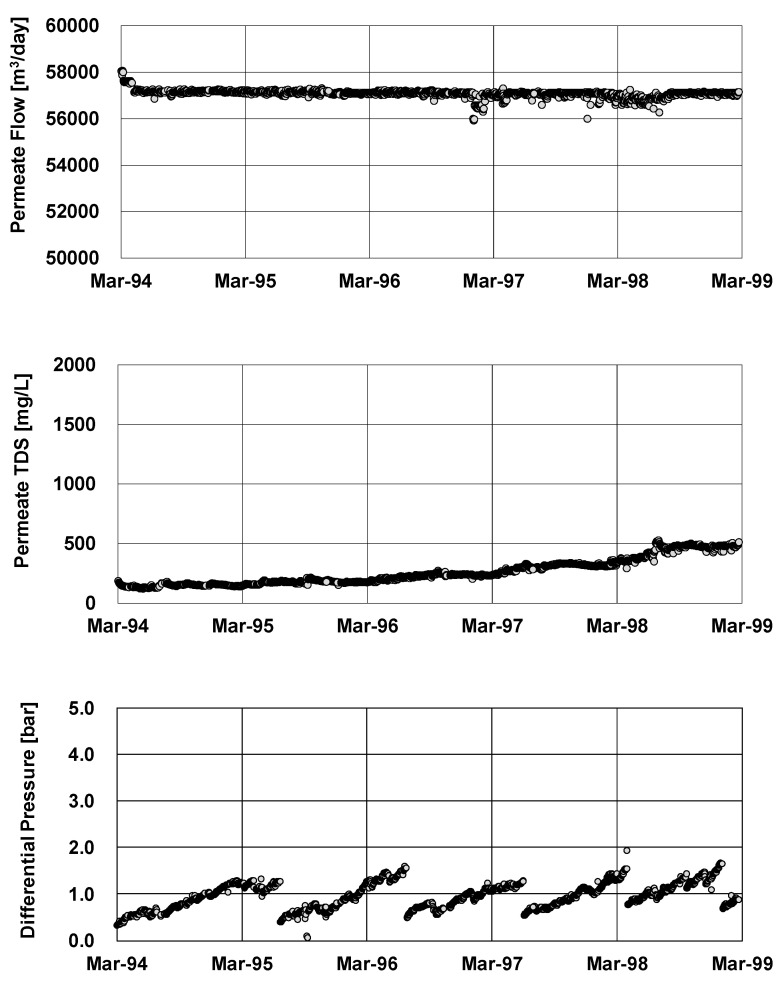
Performance of one of the RO Train in Jeddah 1 phase II plant. Data from [94].

Hypochlorous acid was continuously injected at the intake point in order to suppress the biological growth in the pretreatment systems such as a dual media filter (DMF) and micron cartridge filter (MC). By optimizing the ICI conditions as discussed above, the Jeddah plant has simultaneously prevented both membrane degradation and biofouling. As shown in Figure 16, the Jeddah plant was able to obtain excellent performance during the first 5 years of the operation without any replacement. This 5-year operation at the Jeddah plant was the first successful example of the ICI method in the world. 

**Figure 17:**
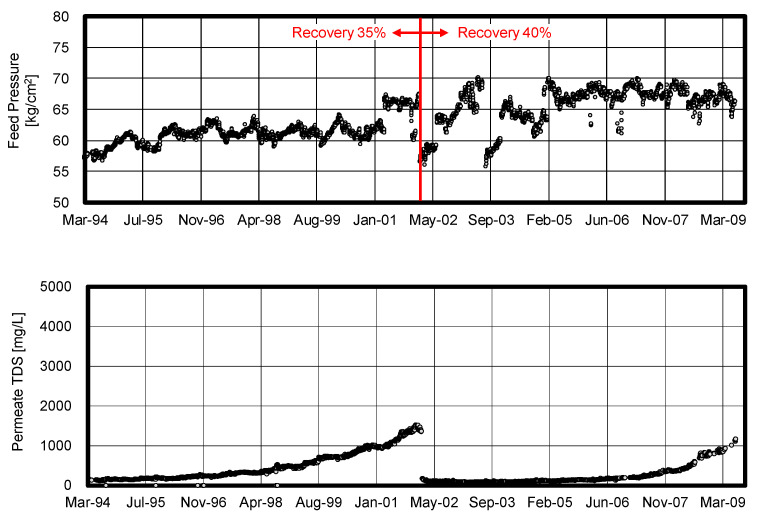
Seawater RO desalination in Jeddah 1 phase II plant. Data from [94].

The Jeddah plant was operated at a recovery ratio of 35.5% initially. Since the actual feed pressure never reached the designed maximum pressure (70 kg/cm^2^) because of the stable operation as shown in Figure 17, the Jeddah plant was able to increase the recovery ratio up to 40.5% successfully without an addition of RO modules [94]. According to this high recovery operation as shown in Table 1, the feed pressure increased by 10.8% and the production increased by 9.2%. Consequently, the product total dissolved solid (TDS) increased at high recovery operation due to a higher concentration in the RO modules. However, the product TDS was still acceptable in this case due to the high salt rejection characteristic of the CTA-HF membrane. The production per feed pressure of a high recovery operation was 3.94 m^3^/h/train/(kg/cm^2^), which was higher than 3.83 m^3^/h/train/(kg/cm^2^) of the original operation. This technique contributed to improving the saving of electrical energy of the high pressure pump.

#### 4.2.2. Ras Al Khair SWRO Plant 

The Ras Al Khair SWRO plant (capacity of the first pass: 899 m^3^/h × 16/train × 24 h = 345,216 m^3^/day; capacity including the second pass: 809 m^3^/h × 16 train × 24 h = 310,656 m^3^/day) is one of the largest membrane facilities ever to be built in the Arabian Gulf, where high salinity seawater and red tides make it difficult to operate a SWRO plant stably [95].

**Figure 18:**
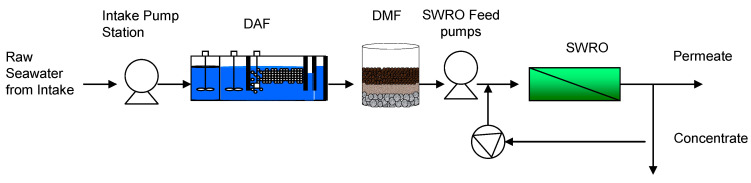
Seawater RO desalination system at the Ras Al Khair SWRO plant. Previously published as part of the International Desalination Association (IDA) World Congress Proceedings, Dubai, 2019 [95].

Raw seawater through an open intake is fed to a dissolved air flotation (DAF) system, and then flows into the DMF as a main pretreatment process before being fed to the first pass SWRO system as shown in Figure 18. In this plant, the ICI method is adopted to eliminate biofouling of the membranes. The designed recovery is set to 35 %, and the pretreated feed seawater is pressurized to about 7 MPa.

The conductivity of raw seawater varied from 61,200 to 66,300 μS/cm. On the other hand, the conductivity of feed water was lower (from 60,500 to 64,000 μS/cm) than that of raw seawater because the brine water of the second pass was mixed with the raw seawater. The 15-min silt density index (SDI_15_) value fluctuated and varied from 2.0 to 4.0 (except in the startup period). The average value of SDI_15_ was 2.8, and this value was considered normal compared with the other desalination plants located by the Arabian Gulf. The residual chlorine concentration of the feed water varied from 0.05 to 0.34 mg/L (except in the startup period), and it was well controlled within the designed criteria (0.2–0.4 mg/L), even though the residual chlorine concentration during the first year since the start-up showed a lower value than 0.2 mg/L frequently. The feed water temperature varied from 15 to 35 °C.

**Figure 19:**
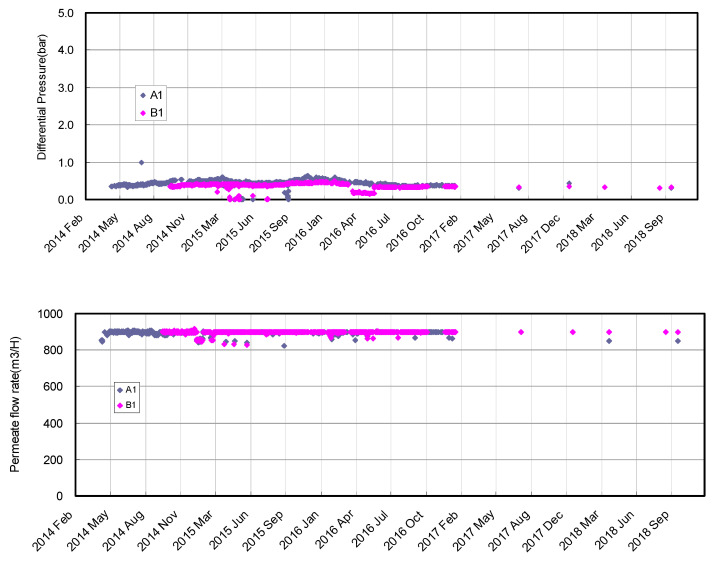

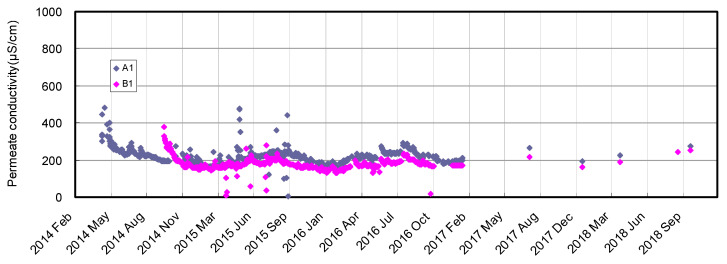
Seawater RO desalination system in the Ras Al Khair SWRO plant [95].

After the initial membrane compaction during the first few months’ operation, no rapid undesired feed pressure increase was observed, and the operation was kept stable for more than 4 years without any membrane replacement or additional chemical cleaning of more than every six months. Figure 19 shows the same operation parameters such as differential pressure (DP), permeate flow rate, and permeate conductivity of Train A1 and Train B1. The DP was also kept low (specifically kept from 0.3 to 0.6 bar) even though no membrane replacement or chemical cleaning was conducted. Therefore, membrane fouling such as biofouling did not occur in spite of the high fouling potential of the Arabian Gulf. It is considered that the pretreatment of raw seawater by a combination of DAF, DMF, and ICI works efficiently to minimize the fouling risk. In addition, the quantity of permeate water was kept stable and satisfied the designed value (900 m^3^/H/train). Moreover, the permeate conductivity was also kept around 200 μS/cm for more than 4 years. Consequently, the Ras Al khair SWRO plant (capacity: 345,000 m^3^/D) achieved stable water production, good product water quality, and high plant availability for more than 4 years in spite of no membrane replacement since the plant startup in 2014. The stable DP in operation and less requirement of chemical cleaning (every six months) from the beginning of operation confirmed the highly effective combination of DAF and DMF for removing mainly colloidal matters in the raw seawater and ICI for preventing biological fouling, even in the Northern Arabian Gulf Seawater.

## 5. Current Development in CTA-HF Membranes

### 5.1. Most Recent CTA RO Membrane

#### Support Layer Design

In recent years, by optimizing the dope composition in the spinning processes of HF membranes to optimize the density structure especially in the support layer, a new CTA-HF membrane has been developed [96]. This newly developed membrane can produce the same amount of water even at a lower feed pressure compared to the conventional CTA-HF membrane used in the seawater desalination plants in the Middle East.

**Figure 20:**
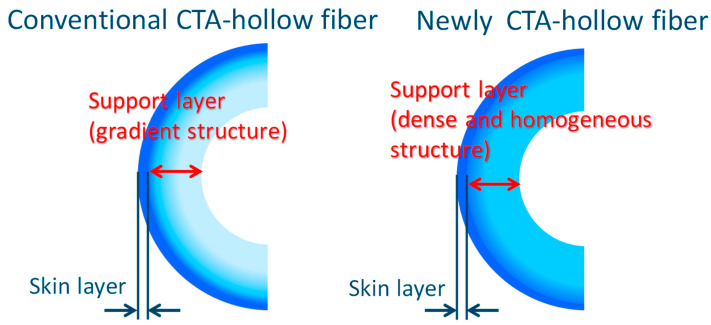
Basic concept of the newly developed CTA-HF membrane.

Figure 20 shows the basic concept of the newly developed CTA-HF membrane. This membrane is basically an asymmetric membrane, but it has a denser and more homogeneous porous support layer than the conventional one without losing the water permeability [96]. In general, it is important to reduce the membrane resistance in order to improve the water permeability of the membrane; therefore, it will be better if the support layer has an asymmetric structure as much as possible to obtain high water permeability. However, interestingly, the newly developed membrane can obtain higher water permeability by optimizing the density balance in the support layer. In addition, the small dispersion of the density distribution in the support layer is expected, which provides performance stability under the high operating pressure for the seawater desalination application.

**Figure 21:**
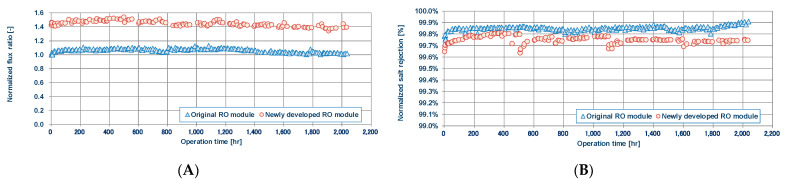
Normalized flux ratio (**A**) and normalized salt rejection (**B**) with conventional RO module and newly developed RO module. Data from [96].

A verification test using 10-in CTA-HF RO modules with the newly developed membrane and the conventional membrane was carried out in a salt water circulation batch RO system to eliminate the influence of fouling under the following operating condition: 35,800 mg/L NaCl, applied pressure of 68 bar, and a recovery ratio of 40% at 35 °C. Figure 21 shows the normalized water permeability of each membrane module with the conventional module water permeability set as 1.0. The results clearly indicate that the new CTA-HF membrane shows about 1.4 times higher water permeability than the conventional one. In addition, salt rejection was sufficiently high with more than 99.5% in both membranes as shown in Figure 21. 

### 5.2. Development of CTA-HF Membranes Targeting Novel Applications 

#### Osmotically Assisted Reverse Osmosis (OARO)

**Figure 22:**
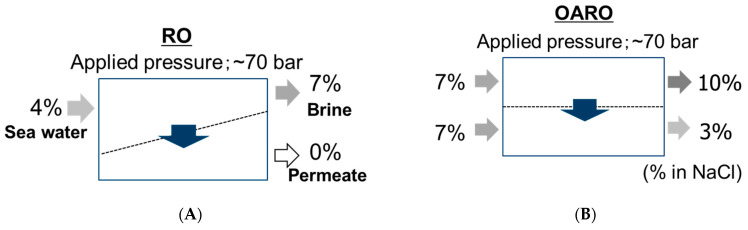
Principle of (**A**) RO and (**B**) osmotically assisted reverse osmosis (OARO) processes.

Recently, CTA-HF membranes have been also used in a novel process for brine concentration, called an osmotically assisted reverse osmosis (OARO) process, in which salinity solutions can be further concentrated up to near its saturated concentration [15,97,98]. The principle of the OARO process is shown in Figure 22. The OARO has also been called CFRO (counter-flow reverse osmosis) [99] and COMRO (cascading osmotically mediated reverse osmosis) [100]. In the OARO process, two solutions of similar or the same salinity are applied to both sides of the membrane in order to diminish the osmotic pressure difference across the membrane, and then a hydraulic pressure is applied as a driving force like the RO process. In this case, the applied pressure can work as a driving force for the water molecule passing through the membrane because the osmotic pressure difference can be diminished and does not impede the driving force, unlike the general RO process case. 

Theoretically, infinite up-concentration can be achieved even when the solutions have much high concentrations where the general RO process cannot be applied to concentrate the solution due to osmotic pressure limitation (>70 bar). In addition, because of the absence of latent heat energy (phase change of water during concentration) unlike evaporation-based techniques such as multistage flash (MSF), multieffect desalination (MED), and mechanical vapor compression (MVC), the OARO process can concentrate the solution up to near its saturated concentration with lower energy consumption. Furthermore, a hybridization of OARO with other pressure-driven process such as RO can be easily installed because of no requirement of an additional high pressure pump by directly connecting the OARO system to the RO system. Therefore, in this case, no additional operational energy is needed for the OARO installation in the absence of a pressure exchanger at the RO system. Timothy V. Barthlomew et al. [98] firstly proposed the OARO as a novel process and theoretically calculated the specific energy consumption (SEC) to compare with the currently available electrically-based brine concentration (BC) technologies such as MVC and RO. Andrew T. Bouma et al. [99] developed a model for CFRO and simulated its performance to propose an energy-efficient multistage CFRO system. Xi Chen and Ngai Yin Yip [100] proposed COMRO and theoretically simulated a SEC and overall system recovery. Jungwon Kim et al. [101] proposed OED-RO (osmotically enhanced dewatering-reverse osmosis), which was somewhat different from OARO, CFRO, and COMRO. The OED-RO processes require a draw solution that has a lower salinity concentration than the feed solution. Therefore, OED-RO can reduce the required hydraulic pressure and also achieve a higher concentration compared with the conventional RO process. 

All the above papers showed the advantages of the OARO process in terms of SEC and achievable concentration. However, the studies were just theoretical calculations using water and salt permeability based on RO and osmotically-driven forward osmosis (FO) performances. Therefore, as an emerging technology, practical membranes specified for the OARO process are highly recommended because the conventional RO and FO membranes are not suitable for the OARO process. For OARO operation, the membranes require compatible features for both RO and FO processes, such as high mechanical strength similar to the RO membrane and a highly porous structure of the support layer of the membrane that can retain the osmotic pressure at the permeate side like FO membrane [102]. However, these features seem to be contrary to each other, and therefore the conventional RO and FO membranes cannot be directly applied to the OARO process.

Most recently, CTA-HF membranes have been applied to demonstrate the OARO application because of its high pressure resistance similar to the RO membranes (>70 bar) and the preferable HF configuration which allows two solutions to be introduced at both sides of the membrane [34,35]. Norihiro Togo et al. performed OARO concentration tests using a pilot-scale CTA-HF membrane module and a theoretical calculation based on the test results. Keizo Nakagawa et al. also performed a multistage OARO concentration test as well as a single-stage OARO concentration test to achieve further up-concentration. In their tests, the maximum number of stages was ten. They practically showed the water flux and achievable concentration at each stage and efficiency of the multistage OARO system. These two studies practically showed the OARO performance using the actual CTA-HF membranes. However, due to the low operating pressure in these studies, the concentration rate was not high since the concentration difference at outlet in the OARO membrane module was highly dependent on the applied pressure. A higher pressure will be better in terms of the concentration rate achieved and will be worse in terms of the electrical energy consumption. Thus, there is a trade-off between the concentration rate and the energy consumption as in the conventional RO operation because OARO is a kind of RO process and the driving force for water movement is related to the hydraulic pressure as well as the osmotic pressure difference across the membrane. There should be an optimal operating condition based on the capital and operation costs, and this will depend on the location such as country because of the difference in electrical energy, civil costs, and so on.

In addition to the above academic research, for the membrane and subsequent module development for OARO application, there are pilot-scale projects announced from companies in the world that target commercializing the technology because a Japanese membrane manufacturer Toyobo has developed a CTA-HF membrane applicable for the OARO process as a commercial product called the BC (brine concentration) membrane [103]. The specifications of the HF BC membrane and the subsequent module (model no. FB10155S3SI) are shown in Table 2. The inner diameter (ID) and outer diameter (OD) are 90 and 200 um, respectively. The effective membrane area per one 10-inch membrane element is about 600 m^2^ with a length of 1400 mm. The CTA-based HF BC module has the same configuration as the CTA-based HF RO membrane module (cross-wound HF configuration, BOE).

Because of the applicable opportunity of the large-scale HF module designed for the BC applications, BC commercialization has been rapidly facilitated at present. In 2019, the Turkish company Hyrec Technologies announced that they signed a memorandum of understanding with the Saline Water Conversion Corporation (SWCC) in the Kingdom of Saudi Arabia to deploy Hyrec’s OARO technology for ZLD purpose [104]. SWCC is one of the biggest companies in seawater desalination, producing 4.6 million m^3^ of fresh water per day in Saudi Arabia. In 2019, U.S. based Gradiant Corporation also announced that they partnered with Saudi Arabian company SAWACO to increase the fresh water production in SWRO using CFRO technology [105]. They were planning to double the fresh water output of SAWACO’s actual SWRO desalination plant by integrating the CFRO to extract more fresh water from the RO brine. The advantage of this process is that it does not require an additional intake or a pretreatment process in order to obtain more permeate water. In addition, in 2020, they announced that their demonstration project was going well and that it met the project’s water quality, efficiency, cost and energy consumption goals. They were planning to scale the process up to 5000 m^3^/D, and the capacity could be up to 15,000 m^3^/D [106]. In 2019, Saudi Arabian SWCC and Toyobo signed an agreement to cover a joint pilot test for reusing RO brine [107]. They performed an OARO pilot study using Toyobo’s commercial-scale BC membrane module and the RO brine in Al-Jubail SWRO desalination plant [108]. Thus, the OARO process using CTA-HF membranes has beneficial potentials in seawater desalination. 

Governments of some countries have already set very strict regulations on waste water discharge to the river and sea for protecting its environment [109]. In addition, an environmentally friendly process is getting more important from the viewpoint of the Sustainable Development Goals (SDGs). Thus, more countries will make regulations on wastewater and brine discharge directly to the environment, and the ZLD and MLD technologies will help to further concentrate the brine for subsequent mining, all of which will be achievable in the very near future. OARO could be one of the best solutions for minimizing the cost and energy consumption as a part of ZLD process compared to the current available thermal and membrane-based technologies. In addition, in seawater desalination, OARO will enable the protection of the environment from brine discharging, the increase of fresh water production (recovery ratio), and opportunities of mining valuable salts such as Mg and Li for sustainable seawater desalination.

## 6. Conclusions

In this paper, recent developments and research trends on the CTA-HF membrane were summarized. The membrane and module, flux theory for the module, and the optimization of plant operation condition are still further developed towards sustainable seawater desalination. The main recent updates are as follows:

1. A high performance CTA-HF RO membrane was developed by designing the support layer structure. The 10-inch module using the developed membrane has about 1.4 times higher water flux performance than that of the previous one, with an acceptable salt rejection performance exceeding 99.5%.
2. To estimate the module performance, a new combining model based on computer fluid dynamic (CFD) and friction concentration polarization (FCP) models were developed, and this new analytical model proved the effectiveness and accuracy of the previous FCP model at the practical level.
3. Optimization of intermittent chlorine injection (ICI) provided the stable and reliable operation in SWRO plant with ensuring safe drinking water quality.
4. In addition to the RO membrane purpose, the CTA-HF membranes were also preferably used as other types of membrane, such as BC.
5. For the brine concentration (BC) application by OARO process, a novel CTA-HF membrane and its 10-inch module have been well developed. Moreover, a hybrid process of RO and BC enables (1) minimization of the brine discharge, (2) increase of the fresh water production (recovery ratio), and (3) the provision of beneficial opportunities for mining valuable salts from the brine in the near future.

With the total global installed desalination capacity still rapidly increasing, securing a reliable and safe water production via seawater RO desalination will become more important in the future. For this purpose, the CTA-HF membranes and their recent updates will be useful for designing sustainable SWRO desalination technology in the future.

## Figures and Tables

**Table 1:** Comparison of 35% and 40% recovery ratio operation in Jeddah RO-2 plant.

Operation	Original Operation	High Recovery Operation
Recovery	35.5%	40.5%
Feed pressure	62.0 kg/cm^2^	68.7 kg/cm^2^
Differential pressure	1.07 kg/cm^2^	1.01 kg/cm^2^
Production	237.3 m^3^/h/train	271.0 m^3^/h/train
Product TDS	365 mg/L	383 mg/L
Date	June 1998	March 2008

**Table 2:** Developed CTA-based HF membrane and 10-inch module specialized for BC applications.

CTA-Based BC HF Membrane			10-Inch BC Module (FB10155S3SI)		
ID	OD	Thickness	Diameter	Length	Membrane area
[μm]	[μm]	[μm]	[mm]	[mm]	[m^2^]
90	200	55	280	1400	600

## Data Availability

Data sharing not applicable.
